# Prognostic Risk Factors for Severe Outcome in the Acute Phase of Neonatal Hypoxic-Ischemic Encephalopathy: A Prospective Cohort Study

**DOI:** 10.3390/children8121103

**Published:** 2021-11-30

**Authors:** Agnese Suppiej, Giovanna Vitaliti, Giacomo Talenti, Vittoria Cuteri, Daniele Trevisanuto, Silvia Fanaro, Elisa Cainelli

**Affiliations:** 1 Department of Medical Sciences, Pediatric Section, University of Ferrara, 44124 Ferrara, Italy; giovanna.vitaliti@unife.it (G.V.); silvia.fanaro@unife.it (S.F.); 2 Neuroradiology Unit, University Hospital of Padova, 35128 Padova, Italy; giacomo.talenti@aopd.veneto.it; 3 Department of Women and Child Health, University of Padova, 35128 Padova, Italy; cuteri.vittoria@gmail.com (V.C.); daniele.trevisanuto@unipd.it (D.T.); 4 Department of General Psychology, University of Padova, 35128 Padova, Italy; elisa.cainelli@unipd.it

**Keywords:** hypothermia, encephalopathy, infant, neurological impairment, neonatal seizure, emergent cesarean section

## Abstract

In the first days after birth, a major focus of research is to identify infants with hypoxic-ischemic encephalopathy at higher risk of death or severe neurological impairment, despite therapeutic hypothermia (TH). This is especially crucial to consider redirection of care, according to neonatal outcome severity. We aimed to seek associations between some neonatal routine parameters, usually recorded in Neonatal Intensive Care Units, and the development of severe outcomes. All consecutive patients prospectively recruited for TH for perinatal asphyxia, born between February 2009 and July 2016, were eligible for this study. Severe outcome was defined as death or major neurological sequelae at one year of age. Among all eligible neonates, the final analysis included 83 patients. Severe outcome was significantly associated with pH and base excess measured in the first hour of life, mode of delivery, Apgar score, Sarnat and Sarnat score, electroencephalogram-confirmed neonatal epileptic seizures, and antiepileptic therapy. Studying univariate analysis by raw relative risk (RR) and 95% confidence intervals (CI), severe outcome was significantly associated with pH (*p* = 0.011), Apgar score (*p* = 0.003), Sarnat score (*p* < 0.001), and Caesarian section (*p* = 0.015). Conclusions. In addition to clinical examination, we suggest a clinical-electroencephalographic protocol useful to identify neonates at high neurological risk, available before rewarming from TH.

## 1. Introduction

Hypoxic Ischemic Encephalopathy (HIE) is a neonatal neurological syndrome characterized by central *nervous* system depression, seizures, and abnormal electroencephalogram (EEG). Reported incidence is 1–2/1000 newborns [1]. It is caused by perinatal asphyxia that leads to metabolic acidosis and low Apgar scores [2,3,4]. The HIE is classified according to the severity of encephalopathy using the Sarnat and Sarnat score, which is based on clinical signs and EEG alterations [5]. The cerebral damage is produced through three different phases: a potentially reversible primary phase, a latency phase, and a phase of irreversible secondary damage leading to cellular death, starting from the 6th to the 15th hour [6]. Before the third phase, a therapeutic window allows intervention with hypothermic treatment. In recent years, several international trials had demonstrated that therapeutic hypothermia (TH) significantly reduces mortality rate and long-term disability when started before 6 h of life and carried on up to 72 h [7,8,9]. In the pre-hypothermia era, a relationship between some neonatal factors, detected in the first hours after birth, and outcome has been well established. However, severe outcomes are still a challenge even after introduction of TH [10,11]. Besides the results of clinical trials, a major focus of research is to verify the validity of early prognostic markers [12]. Particularly, there is a need to identify infants with HIE at higher risk of death or severe neurological impairment, despite TH. This is especially crucial in the first few days after birth also in view of follow-up visits for targeted developmental care.

The more severe sequelae usually manifest within the first year of life and are reliably assessed at one-year follow-up. We report the results of a seven-years single-center prospective longitudinal cohort study aimed to seek associations between some of the neonatal routinely observed parameters and the development of most severe outcomes.

## 2. Materials and Methods

The present study was carried on at the Pediatric University Hospital of Padua, where TH was started in 2009. All 118 consecutive patients prospectively recruited for TH for perinatal asphyxia between February 2009 and July 2016 were eligible for the study. Inclusion criteria for TH adopted at our institution were as follows: (1) gestational age at birth ≥36 weeks, (2) any of the following: arterial umbilical cord or first blood gas analysis (within 1 postnatal hour) with pH < 7.0, and base excess < 12, or 10-min Apgar score < 5, or need for respiratory support at 10 min of life, and (3) moderate to severe encephalopathy within 6 h after birth. For the scoring of neonatal encephalopathy, we used Sarnat and Sarnat criteria [5].

The protocol for TH started as soon as possible after birth or at the time of referral from other hospitals. It consisted of whole-body moderate hypothermia (target temperature 33.0–34.0 °C) for 72 h followed by a rewarming rate of approximately 0.5 °C/h. All patients received Fentanyl infusion throughout TH to prevent discomfort and shivering (1–2 microg/kg/h, with boluses as needed).

The protocol included continuous EEG monitoring from the admission to the end of rewarming, magnetic resonance imaging (MRI) after rewarming and within 15 days of life, and recruitment in a post-discharge standardized neurodevelopmental follow-up [13].

Inclusion criteria for this study were having completed TH and having performed the follow-up protocol at one year of age. Exclusion criteria were having received a diagnosis other than asphyxia, such as congenital malformations or inborn errors of metabolism, as known by the age of one year.

We used for analysis the following neonatal data: gestational age, birth weight, cord pH and BE, pH and BE measured within the first hour of life, mode of delivery (cesarean, vaginal), Apgar score, resuscitation at birth, Sarnat and Sarnat score, EEG-confirmed neonatal epileptic seizures (occurrence of electro-clinical or only electrical seizure pattern as described in medical reports), antiepileptic therapy (use of antiepileptic drugs in bolus, administration of maintenance antiepileptic treatment). We did not analyze data on the EEG background activity because this parameter was variably described in the collected EEG reports and not specifically scored. We also collected the MRI results scored as normal, mild abnormalities, moderate/severe abnormalities [12,13]. Still, they were not included in the statistical analysis because our focus was on early prognostic markers available before the end of TH.

The outcome was defined based on findings at 12 months follow-up. Each evaluation included a medical history and a structured neurological and developmental examination carried out by child neurologists (A.C. and E.T.) and by a child psychologist (E.C.) [14,15]. Information about hearing and visual problems and the occurrence of epilepsy were recorded. Child development was assessed using the Griffiths Mental Development Scales [14], a well-recognized tool for measuring infant mental and psychomotor development in the clinical follow-up, and Amiel Tienson Score [15]. The scales evaluate five specific areas of child development (locomotor, personal-social, hearing and language, eye-hand coordination, performance) and offer a global quotient with a mean of 100 and a standard deviation (SD) of 15.

The outcome was defined as follows: severe in the presence of death, bilateral visual acuity < 1/10, deafness requiring acoustic aids, epilepsy, motor or developmental impairment as resulted by the Griffiths Mental Development Scales (quotient < 70) or by the Amiel Tison; normal/mild in the presence of normal development or of all other minor abnormalities [14,15].

## 3. Statistical Analysis

Continuous data were expressed as mean with SD and median with range (min-max). Categorical data were compared between subjects with a severe outcome and those with not severe outcome using Fisher’s exact test and Chi-square test, while continuous data were compared between the two groups using the Mann–Whitney test. Neonatal parameters (gestational age, birth weight, cord pH and BE, pH and BE measured within the first hour of life, Apgar score, resuscitation at birth, Sarnat and Sarnat criteria) were compared between infants with vaginal delivery and those with cesarean section using the same statistics for categorical and continuous data. A relative risk calculation was estimated to identify independent predictors of severe outcomes among neonatal data, which were statistically significant at univariate analysis. A *p*-value less than 0.05 was considered statistically significant. Statistical analysis was performed using SAS 9.8 software.

## 4. Results

**Figure 1:**
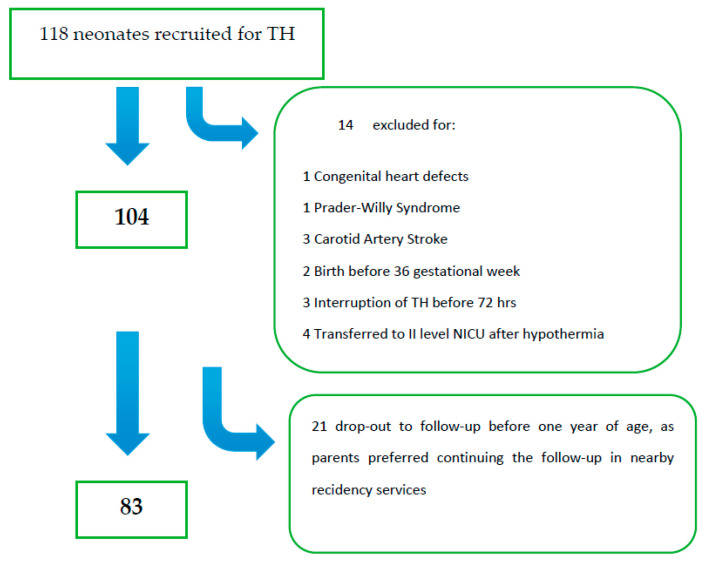
Flow-chart of the study group.

Among 118 eligible patients, 35 were excluded from the initial group (Figure 1); thus, the final analysis included 83 patients. Twenty out of 83 (24%) had an unfavorable outcome; among these, 55% (11/20) died in the neonatal period, and 45% (9/20) developed at least one severe outcome. One (1/9) died at three years of age for respiratory complications in spastic-dystonic severe tetraparesis. Multiple abnormalities consisting of both severe neurological status and associated sensory (visual or auditory deficits) were found in 11/20 (55%).

Seventy out of 83 patients performed MRI: normal in 52 (74.3%) patients, mildly abnormal in 4 (5.7%), and moderately/severely abnormal in 14 (20%).

Urgent cesarean delivery was due to fetal bradycardia in 25 patients (50%), fetal dystocia in 2 (4%), fetal arrest in 1 (2%), abruptio placentae in 9 (18%), prolapsed cord in 2 (4%), twin in 2 (4%), maternal fever in 2 (4%), meconium-stained fluid in 1 (2%) and to two or more of the above factors in 6 (12%). Neonatal parameters were not significantly different in those infants born by cesarean section compared to others, except at 1 min (*p* = 0.001) and 5 min (*p* = 0.01) Apgar score.

Neonatal data are reported in Table 1.

At univariate analysis (Table 1), the severe outcome was significantly associated with pH measured in the first hour of life, mode of delivery (cesarean, vaginal), Apgar score, Sarnat score, EEG-confirmed neonatal epileptic seizures, antiepileptic therapy (bolus and maintenance antiepileptic treatment with IV phenobarbital at a dose of 20 mg/kg bolus and 3–5 mg/kg/die as maintenance dose). Moreover, by studying univariate analysis with raw relative risk (RR) and 95% confidence intervals (CI) on the association between the onset of severe outcome and selected neonatal parameters, we found a statistically significant correlation between the severe outcome and pH (*p* = 0.011), Apgar score (*p* = 0.003), Sarnat score (*p* < 0.001), and Caesarian section (*p* = 0.015) (Table 2).

## 5. Discussion

To date, early outcome markers are very important to promptly identify neonates that will benefit neuroprotective treatment, for management, and counseling to the family. In the present study, neonatal data significantly associated with severe outcomes were emergent cesarean delivery, a severe score of neonatal encephalopathy, and the occurrence of EEG-documented epileptic seizures, particularly without clinical correlate. We found that the severity of encephalopathy had the strongest correlation with the outcome, confirming the previous literature data [7]. Among neonates with severe encephalopathy, 74% had an unfavorable outcome; this finding is, however, better than reported in the pre-hypothermia era when the severe prognosis was seen in 90–100% of neonates with severe encephalopathy [16].

Another risk factor for severe prognosis, found in the present paper, was emergent cesarean delivery, increasing nine-fold the chance of a severe outcome. The prognostic role of emergent cesarean section has not specifically reported in the literature yet. However, cesarean delivery indicates that major obstetrical or fetal problems occurred before birth. Indeed, we found several obstetrical factors leading to emergent cesarean section, such as fetal bradycardia, arrest and dystocia, abruptio placentae, meconium-stained fluid, prolapsed cord, maternal fever, uterine rupture and twinning, which are themselves risk factors for later developmental sequelae. Emergent cesarean delivery is a parameter easily assessed without special expertise. It is interesting that in the setting of perinatal asphyxia and HIE, it heralds a severe prognosis even if the neonate undergoes TH. By contrast, cesarean section was not associated with neonatal comorbidities known as additive risk factors. Neonatal parameters were not significantly different in those born by cesarean section compared to others, except to Apgar score at 1 and 5 min, confirming that this parameter expresses mainly obstetrical risk factors rather than neonatal comorbidities. Among the risk factors, that have been defined for a bad outcome (e.g., hypocapnia, hyperoxia, hypo/hyperglycemia, severity of encephalopathy, latest neonatal seizures and aEEG background voltage activity), the present study confirms the role of severity of HIE, which is the most consistent finding in the literature. Furthermore, it adds a single, useful, and easy to record parameter, the emergent cesarean, which includes several parameters already pointed out as single indicators in the literature.

An important and original finding of the present study is the finding that electroclinical dissociation has a better prognostic role than the occurrence of seizures with a clinical correlate. Electro-clinical uncoupling is typically occurring in the neonatal period as the consequence of brain immaturity, favored by antiepileptic treatment, prematurity, or severe brain damage. It refers to the absence of clinical correlate of an EEG detected seizure, because the epileptic focus is unable to propagate to eloquent brain areas. In our population, 31% of patients had seizures, and 50% of them lacked a clinical correlate. This finding agreed with the current literature showing that among neonates treated with hypothermia manifesting neonatal seizures, half of the seizures are subclinical and cannot be detected without continuous EEG monitoring [17]. However, our data point out the need to perform continuous EEG monitoring in these neonates not only to detect seizures and appropriately treat patients but also for prognostic purposes. The prognostic role of seizures affected by HIE has been debated in the literature [18,19,20,21,22,23,24]. Shah et al. [18] suggest that neonatal seizures increase cerebral damage because they increase metabolic demand and reduce oxygen and nutrient delivery in other cerebral regions; moreover, the increase in excitatory amino acids caused by seizure activity worsens ischemic damage. Indeed, the literature has not completely clarified if seizures themselves may independently increase the risk of a dismal prognosis or if they are only a severity marker of the underlying brain damage.

In this study, we analyzed the outcome separately in only-electrical and electro-clinical seizures and found that only-electrical seizures, lacking a clinical correlate, better predicted the unfavorable outcome. Moreover, we found that patients with severe outcome needed antiepileptic bolus and maintenance IV treatment significantly more often than those with nonsevere outcome. Considering that all patients had phenobarbital IV treatment both in bolus and as maintenance therapy (at the dose of 20 mg/kg bolus and 3–5 mg/kg/die as maintenance dose), this finding may reflect the occurrence of seizures needing treatment better than the only treatment itself and the type of therapy.

## 6. Limits

Nowadays, MRI is considered the gold standard for neurological prognosis in HIE and our data are in line with the literature. We did not focus on the prognostic role of MRI in the present study, which is well established in the literature, because MRI is usually performed after rewarming for logistic and prognostic reasons.

Seizures were found only in 13 patients. In order to confirm the prognostic role of electro-clinical dissociation in neonatal seizures, a larger sample of patients should be studied. Moreover, as EEG records were no longer available when we analyzed all data from a statistical point of view, we could not review the row EEG to calculate the seizure burden. That is one important marker of brain damage severity.

Finally, given the limited number of patients experiencing the outcome, the likelihood of obtaining inflated adjusted estimates was too high, thus we conservatively decide not to perform a formal multivariate analysis.

## 7. Conclusions

This study showed that clinical severity of encephalopathy and occurrence of epileptic seizures without clinical correlate, particularly in the contest of an emergent cesarean delivery, increase the likelihood of death or permanent severe sequelae such as cerebral palsy, epilepsy, deafness, and blindness in neonates affected by HIE. These neonatal risk factors are available to neonatologists before rewarming from TH at bedside without special expertise. We suggest that continuous EEG monitoring should be part of the standard prognostic workup of neonatal HIE. Because TH for neonatal HIE has become standard of care nowadays, it is useful to have a clinical-EEG protocol able to implement anamnestic and biochemical parameters to predict the most severe outcomes.

## Figures and Tables

**Table 1:** Selected neonatal parameters among newborns with versus those without a severe outcome.

	Total (*n* = 83)	Severe Outcome (*n* = 20)	No Severe Outcome (*n* = 63)	*p*-Value
Gestational weeks:				
mean (SD)	39 (1.64)	40 (1.4)	39 (1.7)	0.6476
median (min-max)	40 (36–42)	40 (37–42)	40 (36–41)	
Birth weight (gr)				
mean (SD)	3306.65 (584.5)	3374 (646.2)	3285 (567.4)	0.87
median (min-max)	3300 (1830–4950)	3272 (2400–4950)	3300 (1830–4440)	
Cord Ph	*n* = 49	*n* = 9	*n* = 40	
mean (SD)	6.93 (0.2)	6.89 (0.25)	6.95 (0.2)	0.2768
median (min-max)	6.9 (6.57–7.44)	6.8 (6.57–7.38)	6.93 (6.57–7.44)	
pH first hour	*n* = 75	*n* = 17	*n* = 58	
mean (SD)	6.94 (0.22)	6.82 (0.27)	6.97 (0.19)	0.0023
median (min-max)	6.95 (6.5–7.44)	6.77 (6.5–7.38)	6.96 (6.5–7.44)	
BE	*n* = 41	*n* = 7	*n* = 34	
mean (SD)	−15.56 (7.12)	−17.7 (12.02)	−15.11 (5.83)	0.5103
median (min-max)	−15.3 (−33; −0.3)	−17.7 (−33; −0.3)	−15.2 (−27.4; −1.3)	
BE first hour	*n* = 72	*n* = 16	*n* = 56	
mean (SD)	−19.06 (5.85)	−21.62 (7.11)	−18.33 (5.29)	0.07
median (min-max)	−18.35 (−34; −5.3)	−22.7 (−33; −8)	−18 (−34; −5.3)	
Resuscitation *n*(%)	*n* = 75	*n* =13	*n* = 62	
Yes	65 (86.6%)	13 (100%)	52 (83.8%)	0.11
No	10 (7.5%)	0 (0%)	10 (16.2%)	
Mode of delivery				
Cesarean	50 (60.24 %)	17 (85%)	33 (52%)	0.009
Vaginal	33 (39.75 %)	3 (15%)	30 (48%)	
Apgar score 1 min				
median (min-max)	3 (0–9)	2 (0–4)	3 (0–9)	0.0019
Apgar score 5 min				
median (min-max)	5 (1–10)	4 (1–7)	6 (1–10)	0.0014
Apgar score 10 min				
median (min-max)	6 (1–10)	5 (1–9)	7 (1–10)	0.0204
Sarnat score				
moderate *n* (%)	64 (77%)	6 (30%)	58 (92%)	<0.0001
severe *n* (%)	19 (23%)	14 (70%)	5 (8%)	
Clinical neonatal convulsions *n*.	21 (25%)	9 (45%)	12 (19%)	0.002
Electro-Clinical neonatal convulsions *n*.	13 (16%)	6 (30%)	7 (11%)	0.0429
Electrical Noenatal convulsions *n*.	13 (16%)	7 (35%)	6 (10%)	0.0063
IV bolus of antiepileptic treatement	13 (16%)	7 (35%)	6 (10%)	0.0140
Maintenance antiepileptic treatment	22 (26%)	6 (30%)	16 (25%)	0.0140

**Table 2:** Univariate analysis: raw relative risks (RR) and 95% confidence intervals (CI) on the association between the onset of severe outcome and selected neonatal parameters.

Variables	Raw RR (95% CI)	*p*
Gestational age, 1-week increase	1.13 (0.82–1.57)	0.4
Birthweight, 10-g increase	1.00 (0.99–1.01)	0.6
pH, 10-unit increase	0.34 (0.02–0.47)	0.011
1-min Apgar score, 1-point increase	0.63 (0.48–0.89)	0.003
Sarnat score, 1-point increase	27.1 (7.2–102)	<0.001
Cesarean section vs. vaginal dlivery	5.15 (1.37–19.3)	0.015

## Data Availability

Data are available on request to the corresponding author.

## Abbreviations

BE	base excess
EEG	electroencephalogram
HIE	hypoxic ischemic encephalopathy
MRI	magnetic resonance imaging
TH	therapeutic hypothermia
